# *Kocuria varians *infection associated with brain abscess: A case report

**DOI:** 10.1186/1471-2334-10-102

**Published:** 2010-04-27

**Authors:** Cheng-Yu Tsai, Shou-hsin Su, Yu-Hsin Cheng, Yu-lin Chou, Tai-Hsin Tsai, Ann-Shung Lieu

**Affiliations:** 1 Division of Neurosurgery, Department of Surgery, Kaohsiung Medical University Hospital, Kaohsiung, Taiwan; 2 Department of Internal Medicine, Kaohsiung Municipal United Hospital, Kaohsiung, Taiwan; 3 Department of Nursing, Kaohsiung Medical University Hospital, Kaohsiung, Taiwan

## Abstract

**Background:**

*Kocuria*, established by Stackebrandt et al., previously was classified into *Micrococcus*. Only two species, *K. rosea *and *K. kristinae *are reported to be associated as pathogenic and found with catheter-related bacteremia and acute cholecystitis.

**Case presentation:**

We herein report the first case of brain abscess caused by *Kocuria varians*, a gram-positive microorganism, in a 52-year-old man. Hematogenous spread is the probable pathogenesis.

**Conclusions:**

This report presents a case of *Kocuria varians *brain abscess successfully treated with surgical excision combined with antimicrobial therapy. In addition, Vitek 2 system has been used to identify and differentiate between coagulase-negative staphylococcus.

## Background

*Kocuria spp*. are gram-positive, strictly aerobic microorganisms. Previously they were classified into genus *Micrococcus*, but have now been removed from *Micrococcus *based on phylogenetic and chemotaxonomic analysis [1]. This bacterial cluster consists of nine species and is generally considered to be non-pathogenic commensals that colonize the skin, mucosa and oropharynx. However, they can be opportunistic pathogens in immunocompromised patients, though documented cases of infections are rare. We describe a case which presented as a brain abscess caused by *Kocuria varians*. To our knowledge, this is the first reported case of *Kocuria varians *associated with brain abscess in the English literature.

## Case presentation

**Figure 1:**
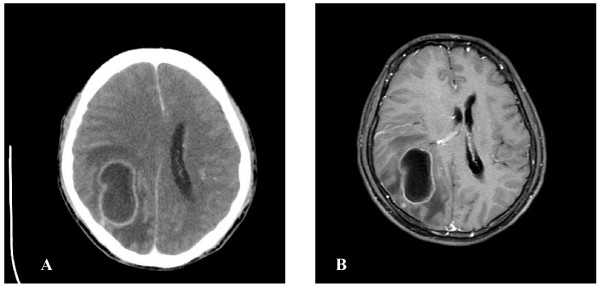
**A. Axial brain CT after contrast medium injection showing a ring-enhancement lesion with perifocal edema over right occipital area**. B. Axial T1-weighted MR image of the brain after gadolinium injection revealed ring-enhancing cystic lesion with mass effect suggestive of right occipital abscess

A 52-year-old male, who had a history of diabetes mellitus and hypertension without regular medication control, presented without fever, but with headache and dizziness for 2 months. These headaches were located over the right occipital area without radiation. The headache worsened while thinking or looking at objects for a long time. Also, he did not have any cutaneous/mucosal lesions prior to or during the period in which these headaches occurred and he had not received any injections or acupuncture. On hospital admission, he still did not have any fever and physical examination revealed normal findings, but a neurological examination showed left side homonymous hemianopia. Brain computerized tomography (CT) (Fig. 1A) and magnetic resonance (MR) (Fig. 1B) showed a right occipital brain abscess. Systemic third-generation cephalosporins (Ceftazidime) and Metronidaole were initially administered pre-operatively for one day by IV. Then, crainotomy and surgical extirpation were performed promptly after a completed image study identified the abscess. The capsule of the abscess was completely removed during the operation. Pus culture yielded a gram-positive microorganism identified as *Kocuria Varians *by the Vitek 2 system. Post-operatively, Metronidazole was ceased due to the outcome of an antibiotic susceptibility test report, for 4 weeks Ceftazidime was administered and then shifted to oral form third-generation cephalosporins (Ceftibuten) for 2 weeks. Neurological function was successfully recovered after operation

### Microbiological diagnosis

**Figure 2:**
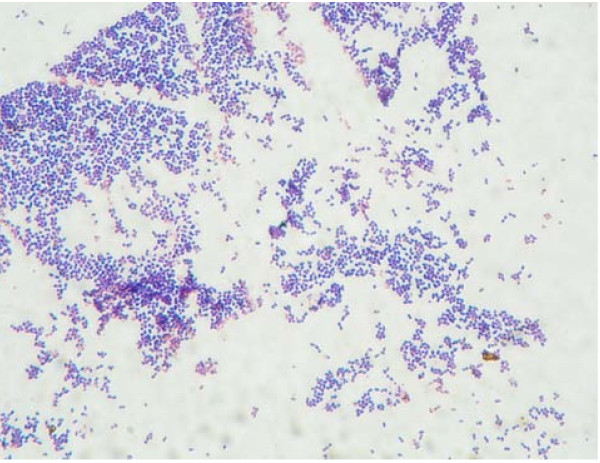
Gram stain revealed gram-positive cocci arranged intetrads

**Figure 3:**
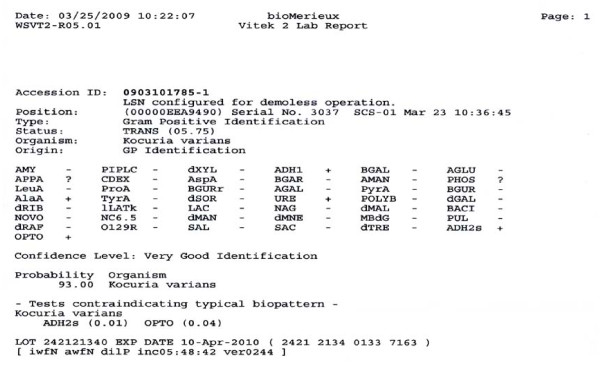
Vitek 2 lab reported organism is *kocuria varians*

Culture of brain abscess was performed with sheep blood agar, eosin methylene blue agar and chocolate agar. The plates were incubated at 35 degrees for 48 hours. Anaerobic culture was performed on Bacteroides Bile Esculin agar and incubated at 35 degrees for 48 hours. Gram-positive cocci arranged in tetrads and non-hemolytic, catalase positive, coagulase negative and nonmotile were found. (Fig 2). Anaerobic culture did not find any anaerobic microorganism activity. Identification was performed using the Biome Rieux Vitek 2 system. The isolate was identified as *Kocuria varians *with very good validity (Fig 3). A useful alternative means of identification, 16s RNA was not performed. The antibiotics sensitivity was performed and sensitive to penicillin, cloxacillin, cefmetazole, clindamycin, Meropenum, penicillin G, Ampicillin/sulbactan, Piperacillin/tazobactam.

## Discussion

The incidence of brain abscess varies between 1500 to 2500 cases/year in the US, with higher incidences in developing countries [2]. It occurs with the following predisposing sources: 1) contiguous spread; 2) hematogenous spread; 3) head trauma; and 4) neurosurgical procedure. According to the previous literature, the most common modes of direct contiguous spread were from the middle ear, mastoid infections and paranasal sinus [3]. But nowadays, hematogenous spread has become the most common source [2]. The organisms that cause brain abscess are typically bacterial in origin. *Streptococcus *and *staphylococcus *are the most frequent organisms and can be isolated from abscesses of all types and at all sites, whereas *Enterobacteriaceae *and *Bacteroides spp*. are isolated from otogenic temporal lobe abscess [4]. In neonates, *Proteus *and *Citrobacter spp*. are the most common organisms. Occasionally *Mycobacterium tuberculosis *as well as fungal infections can act as organisms. *Listeria monocytogenes *and *Burkholderia pseudomallei *are rarely reported [4]. *Micrococcus spp*. associated with brain abscess is mentioned in only two case reports in the English literature [5,8]. In reviewing the English literatures, *Kocuria varians *related to brain abscess has not yet been documented. Our case is the first reported case in which brain abscess is caused by *Kocuria varians*.

*Kocuria spp*. are gram-positive, aerobic microorganisms and found as normal skin flora in humans and other mammals. Infections related to *Kocuria spp*. are uncommon but can be opportunistic pathogens in immunocompromised patients with underlying diseases. *Kocuria spp*. was previous classified as *Micrococcus spp*. Recently, Stackebrant and colleagues made a taxonomic revision of *Micrococcus *spp. and reclassified it in the new genus *Kocuria spp*. (*Kocuria rosea; K. kristinae; K. varians; K. palustris; *and *K. rhizophila ap. Nov*.) [2,6]. The organism *Micrococcus luteus *has been reported as a pathogen in meningitis [7], intracranial abscess [8], arthritis [9], pneumonia [10] catheter-related sepsis in patients with hemodialysis [11], or leukemia treatment [12]. *K. rosea *and *K. kristinae *have been reported as pathogens causing catheter-related bacteremia and acute cholecystitis [13-15]. Due to the normal flora of skin or oropharynx and the reported catheter-related bacteremia of other species, the possible predisposing source of *Kocuria varians *is by hematogenous spread to brain parenchyma.

Due to phenotypic variability, misidentification of coagulase-negative staphylococcus as *Kocuria *by using standard biochemical analysis is common [16]. Therefore the Vitek 2 system has been used, which is scored as being "very good identification" and is reportedly used in both morphotypes [17]. Also the utilization of genotypic assay, such as 16s RNA is used to confirm species identity [15].

Most strains of *Kocuria *and *Micrococcus *were reported to be sensitive to doxycycline, ceftriaxone, cefuroxime, amikacin, and amoxicillin and erythromycin [18], but in consideration of BBB and penetration of the abscess capsule, third-generation cephalosporins should be used. In our case, surgical excision was performed and then third-generation cephalosporins were administered intravenously for 4 weeks and then shifted to oral form third-generation cephalosporins for 2 weeks as an adequate therapy.

## Conclusion

Herein we report the first case of *Kocuria varians *associated with brain abscess in the reviewing the English literature. Because *Kocuria spp*. is found as normal flora and the hematogenous spread of other related species in reported cases, we supposed *Kucuria varians *caused brain abscess by hematogenous spreading. Besides Vitek 2 system, 16s RNA may be additionally used to confirm and thus avoid misidentification as coagulase-negative staphylococcus. Surgical excision combined with third-generation cephalosporins could serve as an excellent standard therapy for *Kocuria varians *brain abscess.

## Competing interests

The authors declare that they have no competing interests.

## Authors' contributions

CT, AL, YC and TT carried out the clinical study of the patient. SS carried out the culture and specific identification of the bacterium. CT drafted the manuscript. All authors read and approved the final manuscript.

## Pre-publication history

The pre-publication history for this paper can be accessed here:

http://www.biomedcentral.com/1471-2334/10/102/prepub
